# Threaded rods versus arthrodesis nail as a static spacer for two-stage revision total knee arthroplasty

**DOI:** 10.1007/s00402-025-05868-y

**Published:** 2025-04-17

**Authors:** Jonas Sina, Daniel Schrednitzki, Shiraz A. Sabah, Andreas M. Halder, Andrew J. Price, Abtin Alvand, Thomas W. Hamilton

**Affiliations:** 1 Nuffield Department of Orthopaedics, Rheumatology and Musculoskeletal Sciences (NDORMS), University of Oxford, Oxford, UK; 2 Clinic for Orthopaedic Surgery, Hand Surgery and Traumatology, City Hospital Zurich, Zurich, Switzerland; 3 Department for Orthopaedics, Trauma, Hand and Reconstructive Surgery, Sana Kliniken Lichtenberg, Berlin, Germany; 4 Department of Orthopaedic Surgery, Sana Kliniken Sommerfeld, Kremmen, Berlin, Germany

**Keywords:** Periprosthetic joint infection, Revision knee arthroplasty, Two-stage revision, Non-articulating spacer, Bone loss, Treatment failure

## Abstract

**Introduction:**

A spacer is required to maintain limb length and alignment and to provide a stable limb for mobilisation in two-stage revision total knee arthroplasty (rTKA) for periprosthetic joint infection (PJI). Static spacers are indicated in cases of massive bone loss, compromised soft tissues, and ligamentous and/or extensor mechanism insufficiency. The aim of this study was to compare the use of Ilizarov rods to arthrodesis nails for static spacer constructs in first-stage rTKA for PJI.

**Methods:**

This was a retrospective cohort study of 40 patients who underwent two-stage rTKA for PJI between 2019 and 2022. Static spacers were used in all cases, constructed from Ilizarov rods 20 patients and nails in 20 patients. Data collected included number of previous revisions, patient age at first revision, comorbidities and identified organisms. Groups were compared based on outcome measures including complications, reoperations, length of stay and re-revision rates.

**Results:**

The use of Ilizarov rods showed higher rates of intraoperative complications (5% vs. 0%), readmissions (55% vs. 5%), and interstage re-operations (50% vs. 10%). Spacer-related complications occurred in 10 of 20 cases (50%) in the Ilizarov rod group, all due to spacer fractures, compared to none in the nail group (0%) (*p* < 0.05). Re-revision rates for infection after the second stage were similar between groups (*p* = 0.75).

**Conclusions:**

The improved safety profile of arthrodesis nails support their use as a temporary static spacer between first and second stage rTKA.

## Introduction

Total knee arthroplasty (TKA) surgery is a highly clinically and cost effective treatment for patients with end-stage degenerative joint disease refractory to non-operative treatment [1]. Whilst rare, periprosthetic joint infection (PJI) remains a challenging complication associated with significant morbidity and mortality [2]. The incidence of PJI after TKA has been reported to be in the region of 1%, but recent evidence suggests that the burden of this complication is increasing, partly due to better diagnosis and partly due to an increase in the absolute number of primary and revision TKAs performed [3–6].

PJI is managed surgically, which may involve debridement, antibiotics and implant retention (DAIR), single or staged revision, arthrodesis or amputation. While the utilisation of DAIR and single-stage revision is increasing [7, 8], two-stage revision is still considered the gold standard technique [9]. Two-stage revision may be indicated when the infection is associated with severe bone loss, compromised soft tissue envelope, or an unstable knee due to ligament instability or extensor mechanism insufficiency. A two-stage approach may also be preferable if the infectious organism is difficult to treat, such as in fungal PJI, or if it is resistant to first-line antibiotics.

In two-stage protocols, an antibiotic-loaded static or articulating spacer is used between stages to provide mechanical stability to the knee joint by preserving limb length and alignment. This allows the patient to mobilize on the limb while facilitating the release of a high local concentration of antibiotics. This approach reduces the risk of toxic systemic side effects [9–13]. The preservation of mobility could perhaps result in a better range of motion after reimplantation [14].

Static spacers are indicated in patients with severe uncontrolled infections, in the setting of massive bone loss and compromised soft tissue coverage over the joint, and where there is concern about the function of the ligaments and/or extensor mechanism. Static spacers are also more likely to be used in repeat revision procedures where previous surgeries may have resulted in massive bone loss due to osteolysis, debridement, or during the process of prosthesis explantation [15, 16]. Despite attempts at salvage and reconstruction, infection may prevail and arthrodesis and above-knee amputation may be required [6, 9, 16–18].

A wide range of static spacers consisting of cement alone or cement with metalwork have been reported in the literature, with the gold standard static spacer yet to be defined [19]. Static spacers are associated with a high complication rate, in part due to the patient population in which they are used, with approximately 1 in 10 patients requiring re-operation prior to the planned second stage [19]. Our institution is a tertiary referral centre specialising in the treatment of PJI, managing around 500 revision cases per year, 95% of which are hip or knee arthroplasties. In patients with PJI and significant bone loss (AORI grade 2 or higher), or those requiring a muscle flap for skin coverage or extensor mechanism reconstruction, our preference is to use a static spacer with diaphyseal fixation in the femur and tibia. Historically we have used cement augmented with Ilizarov rods, but in 2020 moved to using an arthrodesis nail (Endo-Model Knee Fusion Nail– Waldemar Link GmbH & Co. KG, Hamburg, Germany) between the first and second stage revision. The aim of this study is to compare the complication rates, reoperation rates, length of stay, and re-revision rates between Ilizarov rods with arthrodesis nails when used as a temporary static spacer for two-stage rTKA for PJI in a consecutive cohort of patients.

**Fig. 1:**
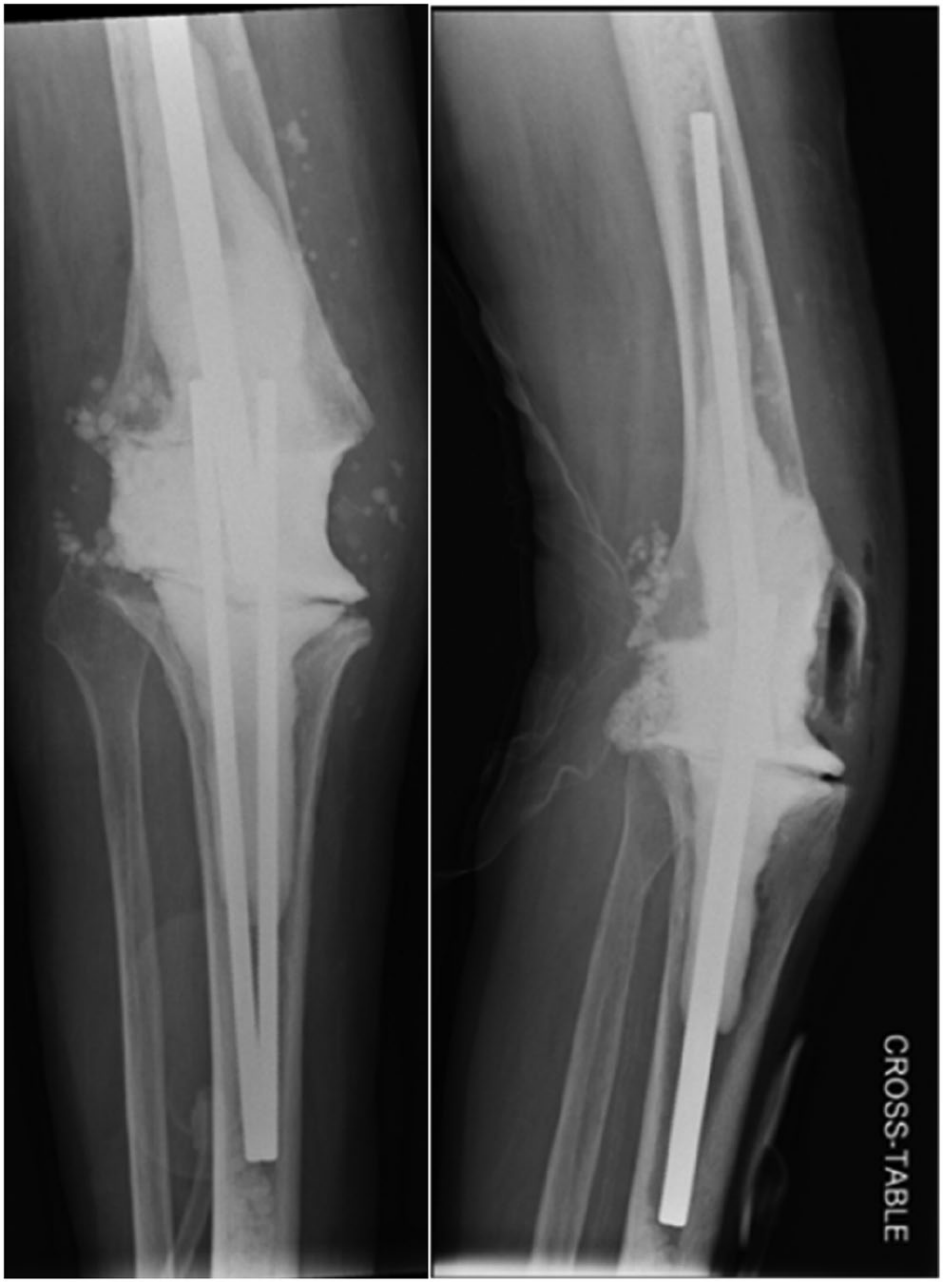
Radiograph in two planes of a static spacer constructed from Ilizarov rods

**Fig. 2:**
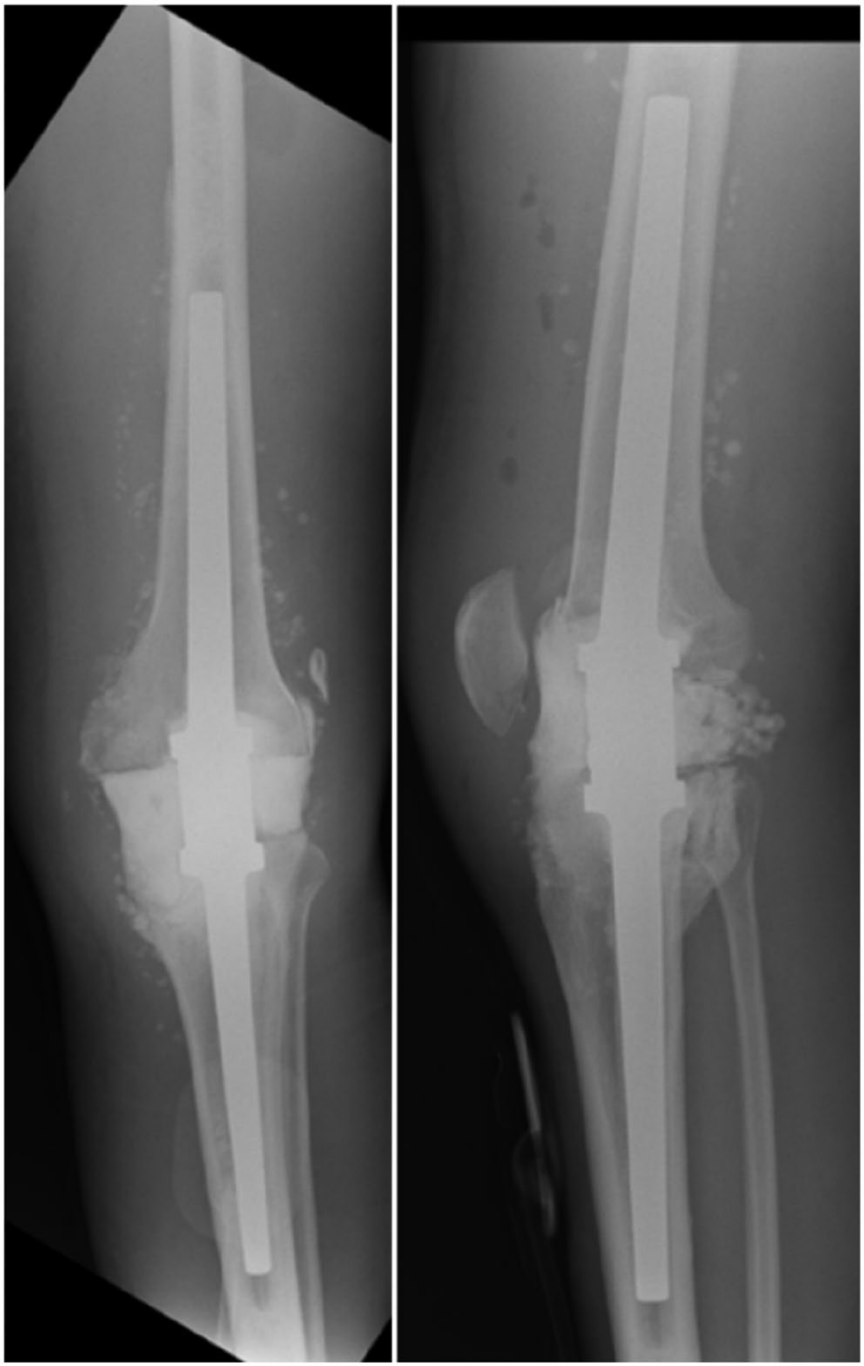
Radiograph images in two planes of a static spacer constructed from arthrodesis nail

Radiographic examples of the static spacer constructed using Ilizarov rods are presented in Fig. 1, while Fig. 2 illustrates the static spacer created with an arthrodesis nail.

## Methods and patients

### Data sources

**Fig. 3:**
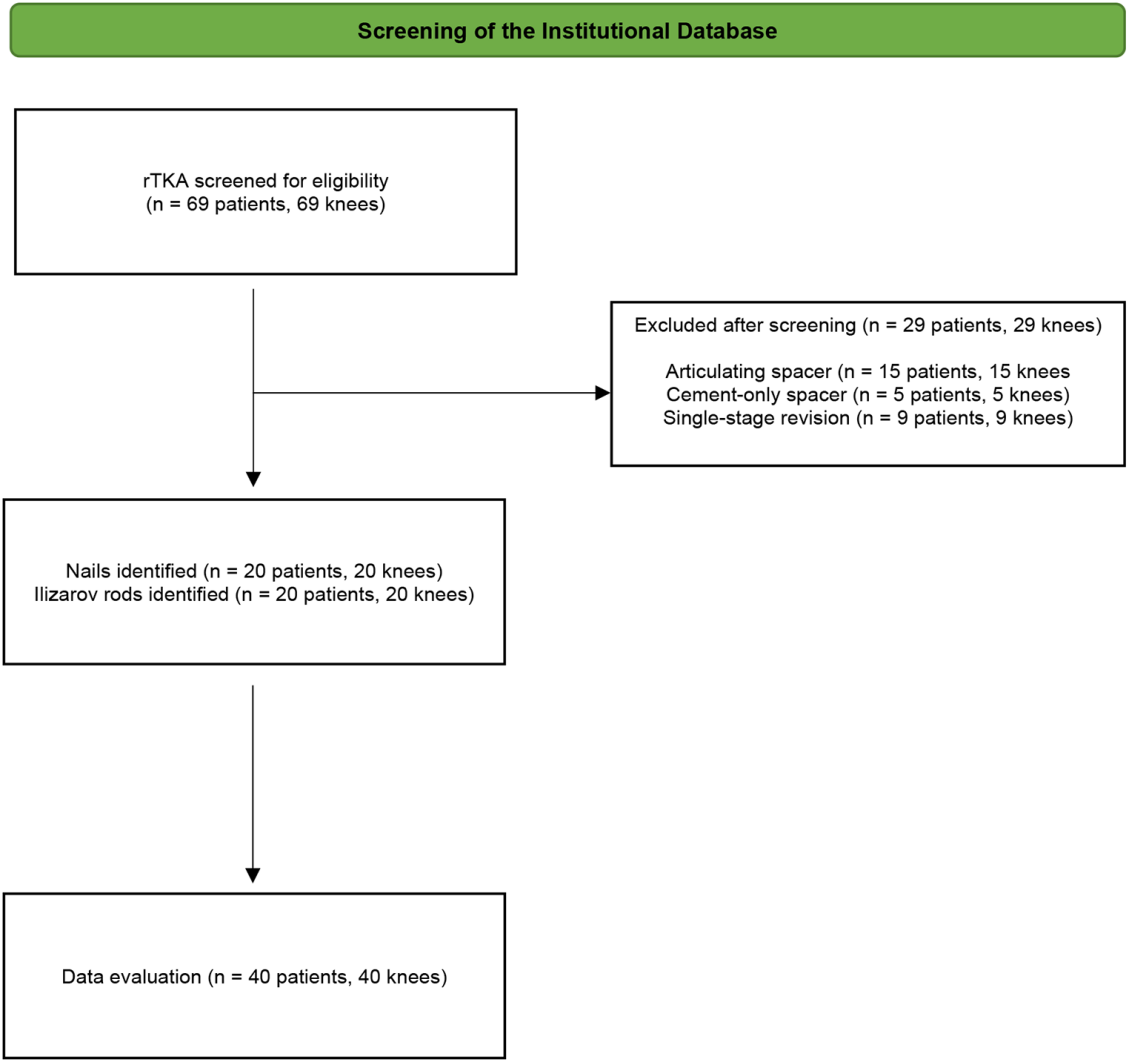
Flowchart

This was a retrospective cohort study within a major revision centre with a specialised Bone Infection Unit. Adult patients (≥ 18 years) who underwent rTKA for PJI, with the intention to treat in multiple stages using one of two types of static spacers in the first stage, were identified from our institutional database between 1 March 2019 and 31 May 2022. A total of 40 consecutive patients were included in the study. In the rod group (*n* = 20), static spacer constructs incorporating cement augmented with Ilizarov rods were used. In the nail group (*n* = 20), static spacer constructs incorporating cement augmented with arthrodesis nails were used. While malignancy was predetermined as an exclusion criterion, no malignancy-related revisions occurred during the study period. A flow chart is shown in Fig. 3.

### Surgical technique

The patients were positioned in a supine position. A sterile washing and draping were performed in the usual manner for all patients. The skin incision was made medially over the knee joint, starting proximally to the patella and extending to the tibial tuberosity, with partial removal of the old scar. The subcutaneous tissue was incised down to the fascia, followed by an arched incision of the medial retinaculum approximately 1 cm parapatellar. Any adhesions within the joint were released, and a subtotal synovectomy was performed. The patella was lateralised, and the knee joint was flexed. Subsequently, the onlay was removed, followed by the removal of the femoral and tibial components after cautious chiselling. All remaining cement residues were then meticulously removed. A total of six tissue samples were collected from various compartments, five of which were submitted for microbiological examination and one for histopathological analysis. A thorough debridement of the joint cavity and both shafts was performed, followed by irrigation using jet lavage.The arthrodesis was performed differently depending on the group.

**Fig. 4:**
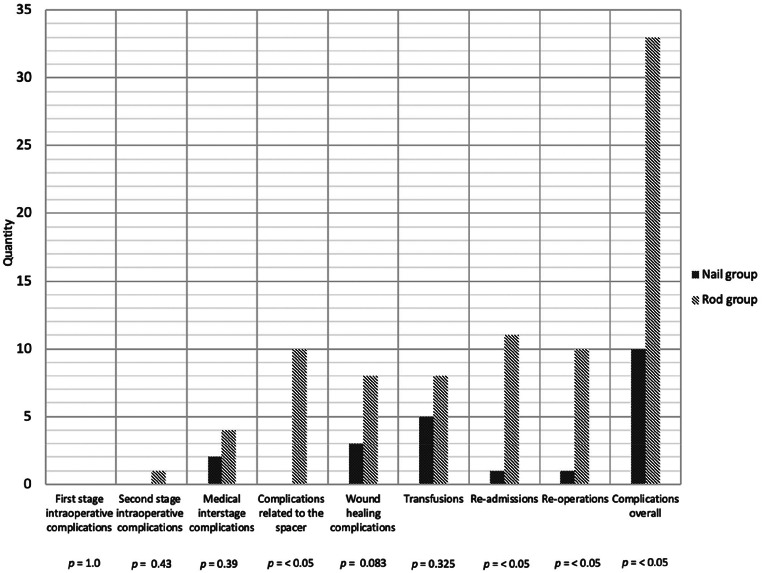
Comparison of complication rates by category: Nail vs. Rod Group

In the rods group, the rods were inserted into both the tibial and femoral medullary canals. Subsequently, the tibia and femur were cemented, and the joint cavity was filled with PMMA.

In the nails group, the shafts were fitted, and their length was determined. The arthrodesis nail was then inserted, followed by a trial coupling and radiographic control. The tibia and femur were then cemented, the coupling module was connected and screwed in place, and finally, a PMMA spacer was inserted into the joint gap.

After the arthrodesis was completed, further irrigation and haemostasis were carried out. The wound was closed in layers, and a sterile compression dressing was applied. Finally, a radiographic control in two planes was performed to check for possible fractures and confirm proper axial alignment of the implanted components.

### Data collection

Patient demographics (Gender, Age, BMI) and clinical information (Comorbidities, Number of previous revisions) were extracted from the institutional database, with additional data collected from the electronic medical record and Picture Archiving Communication System (PACS) as necessary.

### Outcome measures

#### Complications

Intraoperative and interstage complications (including wound healing issues, perioperative bleeding requiring transfusion, spacer-related complications, and hospital readmissions) as well as medical complications (such as thromboembolic events, myocardial infarction, cerebrovascular accidents, pulmonary embolism, acute kidney injury, and death) were identified through a review of patient records and surgical notes.

#### Reoperations

Reoperations between the first- and second-stage revision, including repeated first-stage procedures or early second-stage revisions, were identified by analyzing surgical records and the electronic medical record system.

#### Length of hospital stay

Days elapsed from first-stage revision to discharge after first-stage revision were calculated for each patient, and the median length of stay (LOS) was calculated for each group. Additional days of hospitalisation following readmission after discharge were included.

#### Outcome at latest follow up/further revisions

The outcome of the most recent follow-up was assessed on the basis of whether a further re-revision was necessary after the second stage of revision, or not.

### Statistical analysis

To describe the two study groups, values including means, standard deviations, medians, ranges, and frequencies were used as appropriate. The distribution of the data was assessed using the Shapiro-Wilk test. Significance was tested using the unpaired t-test for normally distributed data and the Mann-Whitney U-test for non-normally distributed data. A p-value of ≤ 0.05 was considered to be statistically significant. All analyses were performed using SPSS^®^ (Version 27).

## Results

### Demographics

The demographic characteristics of the two groups were very similar, with no statistically significant differences observed between them. A detailed summary is shown in Table 1.

**Table 1:** Details on the demographic characteristics of the study population

	All (n = 40)	Rod (n = 20)	Nail (n = 20)	p-value
Gender / n (%)				1.0
* Female*	14/40 (35%)	7/20, (35%)	7/20, (35%)	
Age at first stage / years, median (Q1, Q3))				0.28
* <50*	1/40, (2.5%)	0/20,(0%)	1/20, (5%)	
* 50–59*	7/40, (17.5%)	3/20, (15%)	4/20, (20%)	
* 60–69*	10/40, (25%)	7/20, (35%)	3/20, (15%)	
* 70–79*	14/40, (35%)	9/20, (45%)	5/20, (25%)	
* 80–89*	8/40, (20%)	1/20, 5%)	7/20, (35%)	
* ≥90*	0/40, (0%)	0/20, (0%)	0/20, (0%)	
BMI / mean (SD)	30.94, (6.4)	29.3, (5.24)	32.41, (7.63)	0.15
CCI / mean (SD))	4.4, (1.5)	4, (1.2)	4.9, (1.6)	0.07
No of previous revisions / median(range)	2 (0–4)	2 (0–3)	2 (0–4)	0.82
No of previous revisions / (no. (%))				0.82
* 0*	3/40, (7.5%)	2/20, (10%)	1/20, (5%)	
* 1*	14/40, (35%)	6/20, (30%)	8/20, (40%)	
* 2*	14/40, (35%)	7/20, (35%)	7/20, (35%)	
* ≥3*	9/40, (22.5%)	5/20, (25%)	4/20, (20%)	

### Complications

An overview of the complications and outcomes for both groups is presented in Table 2 and Fig. 4. 

**Table 2:** Details on the complications and outcomes

	All (*n* = 40)	Rod (*n* = 20)	Nail (*n* = 20)	*p*-value
**First stage intraoperative complications (no. (%))**	0/40, (0%)	0/20, (0%)	0/20, 0%)	1.0
**Second stage intraoperative complications (no. (%))**	1/40, (2,5%)	1/20, (5%)	0/20, (0%)	0.43
**Medical interstage complications (no. (%))**	6/40, (15%)	4/20, (20%)	2/20, (10%)	0.39
**Complications related to the spacer (no. (%))**	10/40, (25%)	10/20, (50%)	0/20, (0%)	< 0.05
* Spacer fracture*	7/40, (17.5%)	7/10, (70%)	0/20, (0%)	
* Rod/Nail migration*	1/40, (2.5%)	1/10, (10%)	0/20, (0%)	
* Fracture + migration*	2/40, (5%)	2/10, (20%)	0/20, (0%)	
**Wound healing complications**	11/40, (27.5%)	8/20, (40%)	3/20, (15%)	0.083
**Radiographic complications (no. (%))**	9/40 (22.5%)	9/20, (45%)	0/20, (0%)	< 0.05
**Intra/Perioperative transfusion required (no. (%))**	13/40 (32.5%)	8/20, (40%)	5/20, (25%)	0.32
**Readmissions between 1st and 2nd stage (no. (%))**	12/40 (30%)	11/20, (55%)	1/20, (5%)	< 0.05
**Re-operations (no. (%))**	11/40, (27.5%)	10/20, (50%)	1/20, (5%)	< 0.05
**Re-operations reason (no. (%))**				
* Mechanical failure*	10/11, (90%)	10/10, (100%)	0/10 (0%)	
* Infection*	2/11, (18%)	1/10, (10%)	1/1, (100%)	
* Wound complication*	1/11, (9%)	1/10, (10%)	0/1, (0%)	
**Re-operations type (no. (%))**				
* Repeated 1st stage*	6/11, (55%)	5/10, (50%)	1/1, (100%)	
* Expetited 2nd stage*	5/11, (45%)	5/10, (50%)	0/1, (0%)	
**Length of stay, median (IQR)**	14, (9-22.5)	15, (9-34.5)	13.5, (10–16)	0.40
**Mean follow up, months (range)**	38.2 (16.2–59.8)	47.6, (33.0-59.8)	28.8, (16.2–42.4)	< 0.05
**Outcome at latest follow up**				
* No further revision(s)*	26/40, (65%)	12/20, (60%)	14/20, (70%)	
* Re-revision*	14/40, (35%)	8/20, (40%)	6/20, (30%)	0.75
* Arthrodesis*	0/40, (0%)	0/20, (0%)	0/20, (0%)	
* Amputation*	0/40, (0%)	0/20, (0%)	0/20, (0%)	
* Dead*	2/40, (5%)	0/20, (0%)	2/20, (10%)	
* Lost to follow up*	0/40, (0%)	0/20, (0%	0/20, (0%)	

### Interstage complications

Interstage complications related to the spacer occurred in ten knees (10/20, [50%]) in the rod group corresponding to a spacer-related complication rate of 50%, while no complications related to the spacer were observed in the nail group (0/20, [0%]). In the rod group, spacer fractures were observed in seven knees (7/10, [70%]). In one knee (1/10, [10%]) spacer dislocation/migration was observed. In two knees (2/10, [20%]) spacer fractures with parallel migration of the rods of the spacer construct were observed. A statistically significant difference in the number of spacer-related complications was observed between the two groups (*p* < 0.05).

Spacer failure was managed by expedited second-stage in five knees (5/10, [50%]), repeated first-stage in four knees (4/10, [50%]), and with splinting and continuation of second-stage at the previously planned time in one knee (1/10, [10%]).

All five (5/5, [100%]) patients in whom spacer failure was treated by an expedited second stage completed their full interstage antibiotic plan.

In the rod group, ten knees/patients (10/20, [50%]) underwent further surgery prior to the planned second-stage procedure. Of these knees/patients (10/10, [100%]) required further surgery due to spacer-related complications. In 4 patients/knees (4/10, [40%]) additional complications to the spacer-related complication co-indicated the need for further surgery. In three patients (3/10, [30%]) due to additional wound healing complications and in one patient (1/10, [10%]) due to additional thromboembolic complications. In the nail group, one knee (1/20, [5%]) underwent further surgery prior to the planned second-stage procedure (one (1/1, [100%]) for persistent/recurrent infection).

Interstage wound healing complications were observed in eight out of twenty knees (8/20, [40%]) in the rod group compared to three out of twenty knees (3/20, [15%]) in the nail group. In the rod group, five knees (5/8, [62.5%]) healed with simple wound care, while three knees (3/8, [37.5%]) required surgical treatment. In the nail group, wound complications were managed conservatively in two knees (2/3, [66.6%]), while surgical treatment was necessary in one knee (1/3, [33.3%]).

Interstage medical complications occurred in four patients (4/20, [20%]) in the rod group and in two patients (2/20, [10%]) in the nail group. In the rod group, 3 of the 4 complications (3/4, [75%]) were thromboembolic complications (two PE’s (2/3, [66.6%] and one DVT (1/3, [33.3%])), two of which were managed with medication and one which required surgical treatment by insertion of an IVC filter. One patient (1/4, [25.5%]) was found to have T-wave alterations (ACS) which were treated with medication (DAPT). Of the two medical complications (2/20, [10%]) in the nail group, one (1/2, [50%]) was a DVT that required surgical treatment with angioplasty. One patient (1/2, [50%]) developed SVT, which was treated with adenosine.

Interstage blood transfusion was performed in nine out of twenty patients (8/20, [40%]) in the rod group compared to five out of twenty patients (5/20, [25%]) in the nail group.

### Intraoperative complications

In the rod group, a total of one patient (1/20, [5%]) had an intraoperative complication where a fracture line was noted in the metaphyseal area of the femur when the spacer was removed. The fracture was treated with cerclage wires before proceeding with the second-stage revision and a tibial sleeve was used. There were no intraoperative complications in the nail group (0/20, [0%]).

### Overall complications

Overall, in the rod group, there were 33 complications in 20 knees, with twenty of twenty (*n* = 20/20, [100%]) patients having some form of complication, compared to 10 complications in 20 knees, with seven of twenty (7/20, [35%]) patients who had some form of complication in the nail group. There was a statistically significant difference in the total number of complications between the two groups (*p* < 0.05).

Interstage re/persistent infection was seen in six cases (6/20, [30%]) in the rod group and one case (1/20, [5%]) in the nail group. All cases of re/persistent infection were managed with a repeat first-stage procedure.

### Readmissions/Reoperation

In the rod group, eleven patients (11/20, [55%]) were readmitted during the period between the first and the second-stage revision, compared to one patient (1/20, [5%]) in the nail group. In the rod group, ten (10/11, [90.9%]) were for complications requiring re-operation. Five (5/10, [50%]) expedited second-stage procedures for spacer fractures, four (4/10, [40%]) repeat first-stage procedures for spacer dislocation, fracture or migration and one (1/10, [10%]) repeat plastic procedure for wound healing complications) and there was one readmission (1/11, [9.09%]) for fever managed with broad-spectrum intravenous antibiotics. The readmission in the nail group was due to recurrence of infection (1/1, [100%]) and was managed by repeated first-stage procedure.

A statistically significant difference in the number of hospital readmissions was observed between the two groups (*p* < 0.05).

### Length of stay

The median length of stay after the first-stage procedure in the rod group was 15 days (interquartile range [Q1: 9– Q3: 34.5]). In the nail group, it was 13.5 days (interquartile range [Q1: 10– Q3: 16]) (*p* = 0.40).

### Outcome at latest follow up

Mean follow-up after second-stage surgery was 47.6 months (range 33.0-59.8) in the rod group and 28.8 months (range 16.2–42.4) in the nail group, with a statistically significant difference between the two groups (*p* < 0.05).

At the last follow-up, re-revisions following second-stage implantation had been performed in eight of the twenty (8/20, [40%]) knees treated with Rods. Six of the eight re-revisions were DAIRs (6/8, [75%]) for six persistent/recurrent infections (6/6, [100%]). Two knees (2/8, [25%]) underwent a one-stage revision, one for mechanical failure (1/2, [50%]) and one for mechanical failure with concomitant infection (1/2, [50%]). None of the patients died (0/20, [0%]). In the nail group, six of the twenty knees (6/20, [30%]) had undergone re-revision following second-stage re-implantation. Five of the six re-revisions were DAIRs for persistent infection (5/6, [83%]) and one (1/6, [17%]) two-stage re-revision was for persistent infection with concomitant mechanical failure of the revision prosthesis. Two patients had died (2/20, [10%] after completion of the two-stage process from causes unrelated to the surgical procedure or spacer type. There was no statistically significant difference in the incidence of re-revision for infection following second-stage between the rod and nail groups (*p* = 0.75).

## Discussion

In this retrospective cohort study comparing Ilizarov rods and arthrodesis nails when used as a temporary static spacer for a two-stage rTKA in PJI, several clinically important and statistically significant differences in the outcome measures were identified between the cohorts, all in favour of the nail group. The study demonstrated that the use of Ilizarov rods was associated with higher rates of intraoperative-, postoperative-, and spacer-related complications. Secondly, a higher rate of readmissions, re-operations in the period between the first and second revision stage was observed in the rod group.

Patients undergoing revision KR for PJI comprise a vulnerable group exposed to significant risks, as the procedures are associated with high rates of complications and mortality. Those undergoing a two-stage revision are potentially at higher risk as they are more likely to have massive bone loss, difficult-to-treat infections and multiple previous revisions [20]. A gold standard static spacer has yet to be defined, with previous work revealing a wide variety of static spacer constructs used for two-stage revision KR for PJI [19]. It was found that around 7% of patients undergoing two-stage revision surgery experienced some form of complication necessitating repeat or expedited surgery [19].

Our results demonstrate that spacer-related complications frequently led to expedited second-stage procedures, often requiring urgent or emergency surgery. Such unplanned interventions impose a significant burden on both the patient and the healthcare system. While the optimal duration of antibiotic administration after the first-stage surgery is still under debate [21], proceeding with second-stage surgery prior to the planned completion of antibiotic administration theoretically holds an increased risk of treatment failure. Therefore, identifying the optimal spacer is crucial.

With similar occurrences of VTE and cardiac events, there was no difference in risk for interstage medical complications. However, it was striking that mechanical failure of the Ilizarov rods occurred in 50% of the cases, while there were no cases of mechanical failure when the nail was used.

On the basis of clinical studies that have demonstrated that nails with a diameter larger than 10 mm have a significantly lower risk of fracture [22], previous work already conjectured that the use of hardware with a larger diameter for static knee spacers could lead to a lower risk of mechanical failure [19]. Putting these data in the context of our study, they indicate that Ilizarov rods provide a less stable spacer construct compared to fusion nails. These differences in stability may also account for the differences in wound healing that occurred, as studies have demonstrated that an appropriate biomechanical environment is required for primary wound healing [23, 24]. We observed that with 40%, almost half of the Ilizarov knees had wound healing complications between the stages, frequently requiring re-operation for the wound.

Overall, 100% of patients in the rod group had some form of complication during the course of their treatment and in 50% of all rod patients, the spacers failed mechanically. While the initial cost of Ilizarov rods is considerably lower than that of fusion nails, our study demonstrated that their use is associated with a high risk of failure, predisposing patients to repeated interstage surgery. In consideration of the costs of such complex and resource-intensive interstage re-operations, re-revision surgeries and the extended treatment length required, it is reasonable to conclude that the actual costs of the clinical course when using Ilizarov rods are higher.

The second-stage procedure was expedited in five cases and in four cases a repeat first stage was performed. Several studies reported significantly higher failure rates after re-implantation in cases where appropriate timing for re-implantation was not met or where patients had to undergo a spacer exchange [20–22]. Therefore, it is reasonable to assume that expedited second-stage procedures and repeated first-stage procedures constitute a risk factor for failure following the second stage. However, despite the higher rate of interstage complications and repeated procedures in the rod group, this was not observed in our study.

Kotwal et al. [12] conducted a retrospective study of 58 patients who underwent a two-stage revision for PJI using a static spacer construct consisting of a tibiofemoral intramedullary rod and PMMA. Six of the 58 patients (10%) experienced a complication that required a further or repeat surgical intervention. This rate is comparable to the findings in the nail group (5%), but significantly lower than the rate in the rod group (50%).

In another retrospective study by Skwara et al. [25], patients who underwent a two-stage revision for PJI of the knee joint were examined. The authors used long, stable metal rods and PMMA to construct the static spacer. In 2 of the 21 patients (10%), complications occurred that required a further or repeat surgical intervention. The rate is consistent with the nail group in the present work (5%), whereas the rate in the rod group was markedly higher at 50%.

Hipfl et al. [17] also conducted a retrospective study on patients who underwent a two-stage revision for PJI of the knee joint. They constructed static PMMA spacers (Palacos^®^ R + G; Heraeus, Hanau), which were reinforced intramedullarily with two AO fixator rods made of steel. A complication requiring a further or repeat surgical intervention occurred in 9 of the 97 patients (9%). Again, this rate aligns with those of the nail group (5%), but contrasts sharply with the higher rate observed in the rod group (50%).

In summary, the rate of revision-requiring complications in the nail group of the present work is comparable to the findings reported in other publications. In the study groups of other publications, the rate of revision-requiring complications was approximately 10%, which is broadly consistent with the rate observed in the nail group of the present study, albeit slightly lower at 5%. By contrast, the rate of revision-requiring complications in the rod group of the present study was 50%, which is higher than the rates reported in the study groups of other publications.

We are not aware of any other previous studies that have compared Ilizarov rods with other spacer types and further work is required to define the optimum type of static spacer which may be influenced by patient, disease and surgical factors.

This study is limited by its retrospective design and small number of patients. Demographics and disease outcomes were similar between groups, therefore the groups are comparable. Whilst this report is on a small number of patients, the consistency in outcomes, demonstrating nail to be superior to Ilizarov, is noteworthy and with the results of this study needing to be confirmed in larger, multi-centre studies. It should also be taken into account that the study was conducted during the COVID-19 pandemic, which is why in some cases the period between the first and second-stage may have been longer than planned. Furthermore, it must be considered that the follow-up duration significantly differed between the two groups, with a longer follow-up period in the rod group. However, since the primary focus of this study was on spacer-related complications during the interstage period we believe that this difference in follow-up time does not materially affect the interpretation of the main findings. Nevertheless, the longer follow-up period in the rod group may have influenced the reported re-revision rate and should be considered when interpreting the long-term outcomes.

This study has identified that, compared to arthrodesis nail, the use of an Ilizarov rod cement construct as a static spacer is associated with higher rates of intraoperative-, postoperative- and spacer-related complications, with a higher risk for readmissions and interstage re-operations. The results of the study support the use of arthrodesis nails as a temporary static spacer for between first and second stage rTKA. This study confirms this is a high risk population with 100% percent of patients having a complication following first stage surgery with 50% percent being attributed to the spacer. Consequently, based on the findings of this study, Ilizarov rods are no longer used at our institution. It is important that this information is discussed with patients as part of the shared decision-making process.

## Data Availability

No datasets were generated or analysed during the current study.
